# Relationship between voice impairment and stress coping styles in professionally active teachers

**DOI:** 10.1007/s11845-024-03816-0

**Published:** 2024-10-08

**Authors:** Lidia Nawrocka, Agnieszka Garstecka, Hanna Mackiewicz-Nartowicz, Agata Kozakiewicz-Rutkowska, Paweł Burduk, Anna Sinkiewicz

**Affiliations:** Department of Otolaryngology, Audiology and Phoniatrics, University Hospital No. 2, Collegium Medicum, Nicolaus Copernicus University in Toruń, Ujejskiego 75 Street, 85-168 Bydgoszcz, Poland

**Keywords:** Occupational health, Occupational voice disorders, Prophylaxis, Stress, Teachers

## Abstract

**Background and aim:**

The cause of voice problems in teachers are excessive voice strain, improper voice emission, and wrong emotional responses to occupational stress. The aim of the study was to analyze the relationship between subjective voice assessment of professionally active teachers treated for voice disorders and their stress-coping styles.

**Methods:**

The study included 174 female teachers participating in a 24-day voice rehabilitation program at a sanatorium hospital. The Voice Handicap Index (VHI) self-assessment questionnaire was used to subjectively assess voice impairment. The Coping Inventory for Stressful Situations (CISS) questionnaire was used to assess coping styles. The VHI and CISS questionnaires were administered to the teachers at the beginning of the rehabilitation stay.

**Results:**

Emotion-focused coping was shown to be associated with the subjective assessment of voice dysfunction as assessed by the VHI questionnaire for the total score and all of its dimensions (*p* = 0.04).

**Conclusions:**

In addition to proper voice emission, the prevention and rehabilitation of occupational voice disorders in teachers should also include stress management techniques, as this can help reduce the incidence of functional voice disorders in this occupational group.

## Introduction

Voice disorders are a current public health concern. Teachers are the occupational group most at risk for voice dysfunction [1, 2]. Statistics vary widely, but it is estimated that 10–70% of teachers suffer from voice disorders [3]. Voice dysfunctions are associated with troublesome ailments that adversely impact professional careers [4], reduce job satisfaction, and can even be a reason for leaving the profession [5]. The causes of voice problems in teachers include excessive voice strain [6], improper voice emission [7], chronic stress [8, 9], and inadequate emotional responses to stress and conflict situations [10].

The prerequisite for comfortable voice work in the teaching profession is the ability to tolerate strain on the vocal organ through healthy phonation techniques and adherence to the principles of voice hygiene. Studies show that teachers are aware of the need to take preventive measures to protect the vocal organ and are willing to participate in various forms of voice therapy, both on an outpatient basis and conducted in health resort hospitals [11–13].

The ability to manage stress is an underestimated factor in the prevention of functional voice disorders. A study of Finnish teachers found that stress increased the risk of voice disorders by 3.6 times. The association between voice dysfunction and stress was stronger than that between voice dysfunction and asthma or allergic rhinitis, which are known to be risk factors for voice disorders [1].

The authors of the concept of stress coping styles distinguished three different categories: task-focused, emotion-focused, and avoidance-focused coping [14]. A task-focused coping style involves taking active steps to modify a stressful situation to find a solution. An emotional-focused coping style is characterized by focusing on one’s own experiences and emotions rather than taking effective, rational action and is often favored by those who prefer wishful thinking. The behavior adopted is intended to reduce the emotional tension associated with stressful situations. An avoidance-focused coping style involves turning away from the problem, disregarding any thoughts about the root cause and not engaging in activities to resolve the stressful situation. This style can take two forms: seeking social contact or engaging in substitute activities to divert attention from the existing problem [15].

Task-focused styles are effective in dealing with stress, while emotion and avoidance-focused coping have been shown to be ineffective in dealing with difficult situations [16]. Previous research found that teachers show strong tendencies toward task-focused coping, while emotion-focused and avoidance-focused coping are less frequently observed [17]. Teaching is classified as one of the so-called “helping” professions, in which the full involvement of the teacher’s personality is essential for successful teaching and learning outcomes. In addition to sharing knowledge, teachers must also facilitate the most favorable conditions for student growth [18]. The burden of maintaining high teaching standards and meeting social expectations makes teachers more likely to suffer from occupational stress and burnout [19–21].

The prolonged exposure to stress in the workplace and its effect on the vocal organ shows the importance of monitoring the emotional wellbeing of teachers. Emotional states have been shown to significantly affect the voice production process [22]. The growing incidence of functional voice disorders, associated with pervasive stress and difficulties building interpersonal relationships, calls for further research in this area.

The aim of the study was to analyze the relation between the subjective voice assessment of professionally active teachers treated for voice disorders and their stress-coping styles.

## Material and methods

The study included 174 female teachers between the ages of 27 and 72 (mean age 51) participating in a 24-day voice rehabilitation program at a sanatorium hospital. Ethical approval was obtained from the Ethics Committee of the Collegium Medicum, Nicolaus Copernicus University, and the participants gave informed consent to take part in the study. Based on the initial phoniatric evaluation and treatment records, 139 teachers were diagnosed with hyperfunctional dysphonia, representing 79.89%. Thirty-five (20.11%) had vocal nodules. Of the comorbidities affecting voice quality, laryngopharyngeal reflux was found in 94 teachers (45.98%) and thyroid disease in 37 (21.26%). Questionnaires on subjective assessment of voice dysfunction and stress coping styles were administered to the teachers at the beginning of the rehabilitation stay.

Subjective assessment of each teacher’s voice was carried out using the Voice Handicap Index (VHI) Self-Assessment Questionnaire in the Polish adaptation by Pruszewicz [23, 24]. The VHI test consists of three subscales:

- I—Self-assessment of functional state
- II—Self-assessment of emotional state
- III—Self-assessment of physical state

The VHI questionnaire contains a total of 30 questions, 10 for each subscale. Questions referring to functional state investigate how voice impairments affect daily social and professional life. Questions from the emotional part probe the patient’s view of his or her own voice. The third section of the questionnaire addresses physical issues related to the vocal organ.

In each of the subscales, the respondent can score from 0 to 40 points, with a total of up to 120 points. A range of 0–30 points is considered to be a mild voice impairment, a 31- to 60-point score indicates a moderate voice impairment, and 61 to 120 points is regarded as a severe voice impairment.

Szczepaniak’s Polish adaptation of the Coping Inventory for Stressful Situations (CISS) questionnaire, developed on the basis of the concept by Endler and Parker, was employed to analyze coping styles [25]. This measurement tool is comprised of 48 statements about activities adopted in stressful conditions, each with a response rate of 1 to 5. The results are presented in three scales: task-oriented (TOC), emotion-oriented (EOC), and avoidance-oriented coping (AOC) style. The latter can take two forms: engaging in substitute activities (EISA) and seeking social contact (SSC).

TOC scale—the task-oriented style of coping with stress—involves taking action. People who score high on this scale tend to make an effort to solve the problem. This is an effective style for coping with difficult situations.

EOC scale—the emotion-oriented coping—involves focusing on oneself, on one’s own emotional experiences, which tends to increase tension rather than decrease it in a stressful situation. This style is not conducive to dealing with difficult situations.

AOC scale—the avoidance-oriented style—involves refusing to think about and experience a difficult situation. Instead of seeking a solution to the problem, the person engages in substitute activities such as watching television, overeating, sleeping, thinking about pleasures, or seeking social contact.

Each of the TOC, EOC, and AOC scales consists of 16 items. Subjects can score from 16 to 80 points on each. The EISA subscale has 8 questions and the respondent can score from 8 to 40, while the SSC subscale has 5 items and scores from 5 to 25. The remaining three questions are part of the AOC scale and are not included in the subscales. Scores on each scale are calculated separately according to the key [25]. Parametric descriptive statistics such as arithmetic mean, standard deviation, minimum, and maximum were used to statistically describe the collected material. *R*-Spearman rank correlation was used to examine relationships.

## Results

The job seniority of the surveyed teachers ranged from 2 to 49 years, with an average of 27.7 years. Eighty percent were teachers with more than 20 years of work experience. Based on the initial phoniatric examination and treatment records, 139 teachers were diagnosed with hyperfunctional dysphonia, which accounted for 79.89%. Thirty-five (20.11%) had vocal nodules. Among the comorbidities that have an impact on vocal quality, laryngopharyngeal reflux was found in 94 teachers (45.98%) and thyroid disease in 37 (21.26%).

### Subjective assessment of voice impairment on the VHI scale

**Fig. 1:**
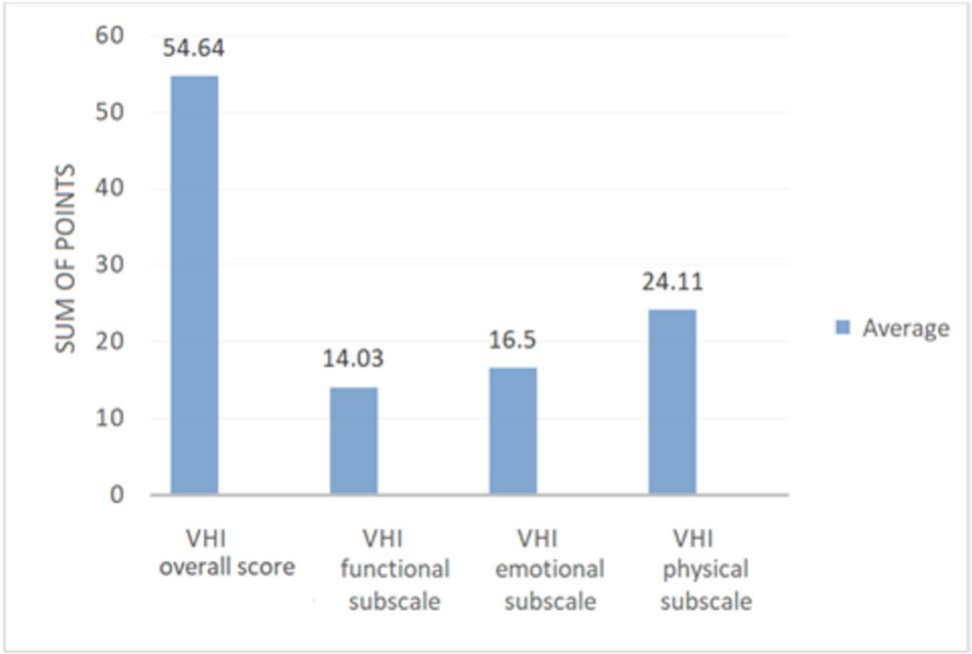
Self-assessment of voice impairment on the VHI scale. Note: The chart illustrates the distribution of scores obtained on the overall VHI scale and its individual subscales

The subjects had an average VHI score of 54.64 points, and the score range was 108 points (min. 7 points–max. 115 points). Patients reported the highest average level of voice impairment on the physical subscale (24.11). Much lower average scores were obtained on the functional (14.03) and emotional (16.5) subscales (Fig. 1).

### Stress coping styles and self-assessment of voice impairment on the VHI scale

The task-focused stress coping strategy ($\overline X$ = 54.29) was predominant. Avoidance-focused coping ($\overline X$ = 48.0) was stronger than the emotion-focused style ($\overline X$ = 43.14). Among the behaviors characteristic of avoidance coping, the subjects more frequently chose substitute activities rather than seeking social contact. The stress-coping styles of the surveyed teachers are shown in Table 1.

**Table 1:** Stress coping styles of the subjects

Types of stress coping styles	*N*	$\overline X$	Minimum	Maximum	SD
Task-focused	174	54.29	34.00	68.00	5.68
Emotion-focused	174	43.14	16.00	69.00	9.42
Avoidance-focused	174	48.00	30.00	70.00	7.28
Substitute activities	174	21.79	13.00	34.00	4.07
Social contact	174	17.90	7.00	25.00	3.33

*SD* standad deviation, $\overline X$ arithmetic average

The relationship between subjective assessment of voice impairment and stress coping styles was analyzed. A correlation analysis was performed for the individual subscales of the VHI self-assessment test.

The correlation coefficients obtained showed a statistically significant connection between all dimensions of the self-assessment of voice impairment. A statistically significant relationship was also confirmed between the overall subjective assessment of VHI voice impairment and the emotional style of coping with stress. In this case, the coefficient of statistical significance was 0.04 (Table 2).

**Table 2:** Relationship between the teachers’ self-assessment of voice impairment on the VHI scale and stress coping styles

*N* = 174 *R*-Spearmans					
VHI subscales	Task-focused style	Emotion-focused style	Avoidance-focused style	Substitute activities	Seeking social contact
I—Functional	0.1026	0.1731	− 0.0137	0.0402	− 0.1475
	*p* = 0.178	***p* = 0.022**	*p* = 0.858	*p* = 0.599	*p* = 0.052
II—Emotional	0.1239	0.1925	− 0.0220	0.0362	− 0.1270
	*p* = 0.103	***p* = 0.011**	*p* = 0.773	*p* = 0.636	*p* = 0.095
III—Physical	0.1231	0.1509	0.0610	0.0750	− 0.0134
	*p* = 0.105	***p* = 0.047**	*p* = 0.424	*p* = 0.325	*p* = 0.860
I–III total	0.1437	0.1531	0.0070	0.0244	− 0.1070
	*p* = 0.058	***p* = 0.043**	*p* = 0.926	*p* = 0.748	*p* = 0.159

*p* signifance level

## Discussion

The relationship between emotional factors and voice disorders in teachers is rarely addressed in the scientific literature. Most publications focus on the analysis of the influence of social and health factors on the development of voice dysfunction in this professional group. Our research investigates the impact of stress coping styles on teachers’ subjective assessment of vocal impairment.

Self-assessment of the patient’s voice impairment is particularly important in the comprehensive diagnosis of voice disorders in teachers [26, 27]. As a diagnostic tool, the VHI provides information that is complementary to the laryngovideostroboscopic examination and the acoustic analysis [28, 29]. The VHI is currently the most widely used voice self-assessment scale [30–34]. In the present study, professionally active teachers had an average rating of 54.6 points for their voice impairment on the VHI scale. The evaluation of physical state indicated that teachers experienced the strongest vocal limitations in this domain (24.11 points), while the emotional and functional assessments registered significantly lower stores (16.5 and 14.03 points, respectively). In studies of healthy people in the general population, the average VHI scores range from 8.9 and 10.5 points [35], and in patients with dysphonia, the average VHI scores range from 35.5 to 48.1 points [36–38]. Ohlsson showed in a group of women diagnosed with voice disorders that the average VHI score was 33, while women without voice disorders had an average score of 10 [39]. In our study, the average VHI self-assessment score was higher compared to the analyses of other authors [37, 38, 40], as the study group consisted of teachers with diagnosed voice disorders who took part in a 24-day voice rehabilitation program at a sanatorium hospital.

In the psychological assessment that took into account the subjects’ stress-coping styles, task-focused coping was clearly dominant. This means that when faced with a stressful situation, most teachers employ problem-solving techniques. The task-focused approach is beneficial in treatment and rehabilitation, as patients are more likely to follow medical recommendations [41]. Emotion and avoidance-focused coping styles are less effective in dealing with job stress. Among the teachers studied, avoidance-oriented coping, in the form of engaging in substitute activities, slightly outweighed the pursuit of social interaction. Avoidance activities included behaviors that did not affect voice quality. Emotions play a more important role in voice work [22].

The emotional style of coping with stress in the teachers studied corresponded significantly with the overall VHI scale and all of its subscales. Individuals who exhibit this style tend to over-experience failure and negative emotions. Inadequate emotional responses to occupational stress have a negative impact on voice quality and endurance, as confirmed in studies by Ma, Dietrich, and Tao [42–44].

The association of emotional lability, understood as a tendency to neurotic reactions in difficult situations, with the development of functional dysphonia and vocal nodules has been confirmed in a study by Roy [45]. Baker proved that although dysphonias can develop after a viral infection, they are most often associated with an emotional or psychological experience of “conflict over speaking out” [46]. In a study by Sanne et al., teachers have been found to have higher levels of anxiety and depression compared to other occupational groups [47].

In analyzing the relationship between emotions and the voice, attention should also be paid to changes in the voice due to menopause. The emotional vacillation, irritability, tearfulness, difficulty concentrating, and sometimes even depression, characteristic of this period, exacerbate voice problems [48]. Post-menopausal women are much more likely to demonstrate symptoms of nervousness and lowered mood [49]. A Vaca study found that people over the age of 50 were more likely to have voice problems [50]. The results of studies by Lin et al. and de Medeiros et al. showed that female teachers had a 1.6-fold higher risk of voice disorders than male teachers [5, 51].

Preparation for voice work should include training in stress management. This problem is overlooked by teachers who focus on didactic and educational tasks, often with excessive anxiety and involvement of their own emotions. This indicates the need to include training in adequate emotional responses to stress along with vocal endurance training for teachers.

## Conclusions

The emotional style of coping with stress influences the subjective assessment of voice impairment in all its dimensions. In addition to working on healthy voice production, stress management techniques should also be included in the prevention and rehabilitation of occupational voice disorders in teachers, since this may help reduce the incidence of functional voice disorders in this professional group.

## Data Availability

All data used to support the findings of this study are available from the corresponding author upon request.
